# Sporotrichosis: Using scanning electron microscopy to decipher the “blackish‐red dot sign” observed under dermoscopy

**DOI:** 10.1111/srt.13775

**Published:** 2024-05-29

**Authors:** Yuan Chen, Lina Li, Zhi Zhang, Runyan Gao, Xin Ran, Jitong Sun, Chaoliang Zhang, Xinyao Liu, Yuping Ran

**Affiliations:** ^1^ Department of Dermatovenereology West China Hospital Sichuan University Chengdu China; ^2^ Academician Workstation of Wanqing Liao West China Hospital Sichuan University Chengdu China; ^3^ Department of Dermatovenereology Henan Provincial People's Hospital (People's Hospital of Zhengzhou University) Zhengzhou China; ^4^ State Key Laboratory of Oral Diseases West China Hospital of Stomatology Sichuan University Chengdu China

Dear Editor,

Sporotrichosis is a chronic infection caused by the *Sporothrix schenckii complex*, infecting the skin, subcutaneous tissues, mucous membranes, lymphatic, and even visceral systems. The presence of the “blackish‐red dot sign” is a characteristic finding commonly observed under dermoscopy in skin lesions of chronic subcutaneous fungal infectious diseases.^1^, ^2^, ^3^, ^4^ Here, we report a case of sporotrichosis with dermoscopic features of the “blackish‐red dot sign” which were observed using scanning electron microscope (SEM).

A 56‐year‐old male patient presented with erythema and nodules in the right facial region, which had been present for one year. Upon physical examination, an erythema measuring 4 cm × 5 cm in size was observed on the right side of face. A subcutaneous nodule measuring 1 cm in diameter can be palpated at the right mandibular angle. The surface of the erythema lesion appeared uneven, with sporadic “black dots” (Figure 1A).

**FIGURE 1:**
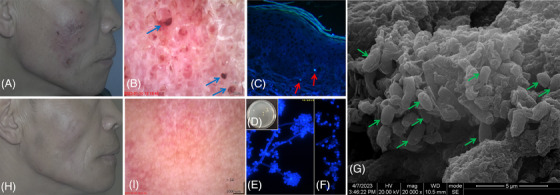
(A) Clinical manifestation of the patient observed for the first time and at follow‐ups. The patient had developed erythema on the right side of face. (B) Dermoscopy of the lesion on initial treatment showed blackish‐red dots (× 61) (blue arrows). (C) Tissue biopsy shows bright blue yeast cells in the dermis (calcofluor white staining, × 400, red arrows). (D) Gray‐white colonies (SDA 28°C for 7 days). (E) The microscopy of the slide culture showed fungal hyphae appearing slender, with spherical conidia arranged in a cluster, forming a plum blossom‐like pattern under the 25°C (SDA for 7 days, × 1000), (F) A significant number of yeast cells with a spherical shape under the 37°C (SDA for 7 days, × 1000). (G) SEM of the “blackish‐red dot sign” revealed a numbers of yeast cells (× 20,000, green arrows). (H, I) After five months of treatment with oral itraconazole, the lesions significantly improved and “blackish‐red dot sign” disappeared.

Under dermoscopy of the erythema lesion, we observed white and pink background and sporadic blackish‐red dots (Figure 1B). This patient was diagnosed with lymphangitic‐type sporotrichosis based on a subcutaneous nodule at the right mandibular angle, a primary cutaneous lesion in the right facial region that has spread along the submandibular lymphatic vessels.^5^ A biopsy from the right mandibular angle subcutaneous nodule revealed purulent granulomatous response in the dermis (Figure S1). Tissue with Grocott's methenamine silver (GMS) and periodic acid‐Schiff (PAS) staining, and acid‐fast staining were all negative. Staining tissue with calcofluor white (CFW) highlight bright blue yeast cells at the dermis level (Figure 1C). Biopsy tissues were inoculated onto Sabouraud Dextrose Agar and incubated at 28°C, developing into gray‐white colonies after seven days. Microscopic examination of the slide culture revealed slender fungal hyphae with spherical conidia arranged in a cluster, forming a plum blossom‐like pattern at 25°C after 7 days (Figure 1E). And yeast cells with a spherical shape were observed at 37°C after 7 days (Figure 1F). The SEM images of slide culture can be found in the supplementary materials (Figure S2). *Sporothrix globosa* (GenBank accession number PP116103) was identified through PCR amplification using Internal Transcribed Spacer (ITS) 1/4 primers, followed by sequencing analysis. Under the guidance of dermoscopy, the blackish‐red dots from the facial lesion were meticulously picked and subsequently observed with SEM after fixation, dehydration, and vacuum drying, demonstrating yeast cells. The patient was administered oral itraconazole 200 mg twice daily, topical naftifine‐ketoconazole cream, and local thermotherapy (42°C) for 6 months, leading to complete healing without recurrence during the 5‐month follow‐up. Dermoscopy showed the disappearance of the “blackish‐red dot sign” (Figure 1H, I).

In conclusion, we report a lymphangitic‐type sporotrichosis case caused by *S. globosa* and diagnosed through mycological examination. Yeast‐like cells were detected in the tissue biopsy using calcofluor white staining, demonstrating greater sensitivity than either PAS or GMS staining methods. Dermoscopy demonstrated the “blackish‐red dot sign” and SEM images showed numerous yeast cells in stereoscopic mode. Previous cases^1^, ^2^, ^3^, ^4^ suggest that the “blackish‐red dot sign” is an inflammatory response that expels pathogenic fungi. Further research is necessary to explore its potential as a dermoscopic clue for diagnosis and therapeutic effective evaluation, more clinical cases are required to investigate this sign effectively.

## CONFLICT OF INTEREST STATEMENT

The authors declare no conflicts of interest.

## Supporting information

Supporting Information

## Data Availability

Data sharing not applicable to this article as no datasets were generated or analyzed during the current study.
