# Supplementary material for: Sporotrichosis: Using scanning electron microscopy to decipher the “blackish‐red dot sign” observed under dermoscopy
Source: Skin Res Technol. 2024 May 29;30(6):e13775. doi: 10.1111/srt.13775 (PMC11135623; doi:10.1111/srt.13775)
Supplement: Supplementary file 1 — Supporting Information [file SRT-30-e13775-s001.docx]

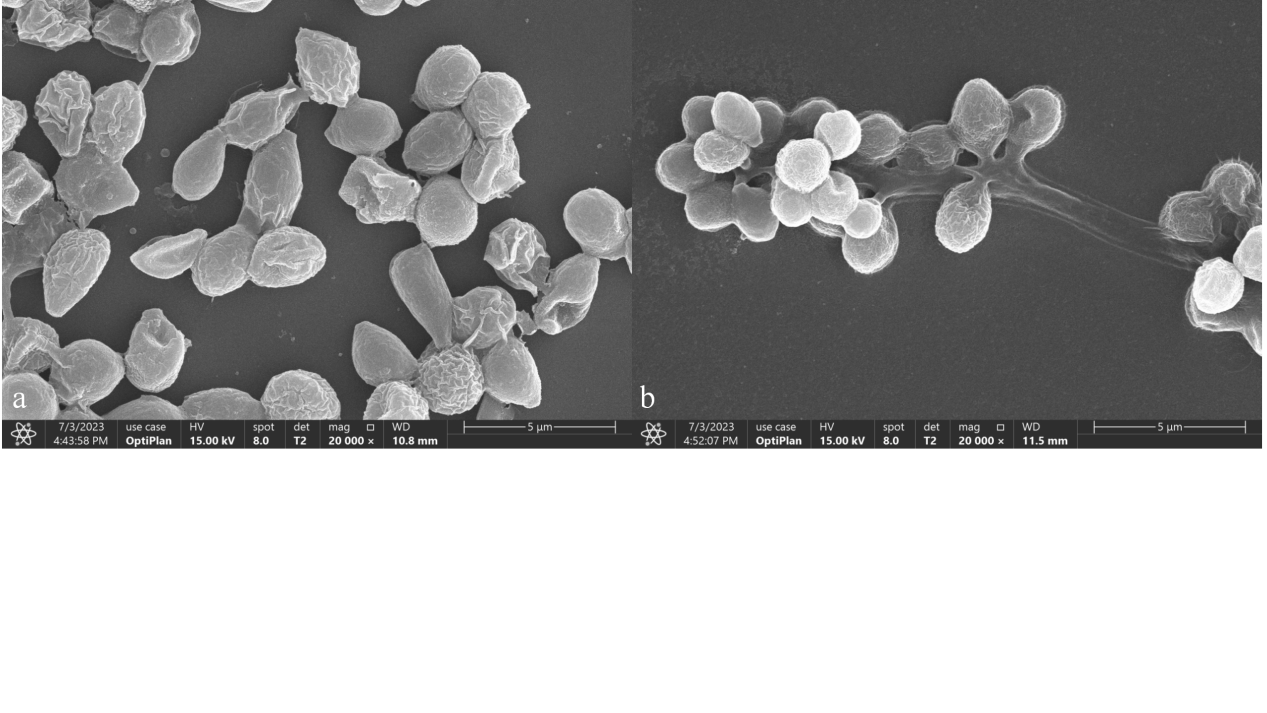


Fig. S1 a: Scanning electron microscope image of the slide cultured fugal yeast cells at 37°C (SDA for 7 days). b: Scanning electron microscope image of the slide culture, revealed slender fungal hyphae with spherical conidia arranged in a cluster, forming a plum blossom-like pattern at 25°C (SDA for 7 days).


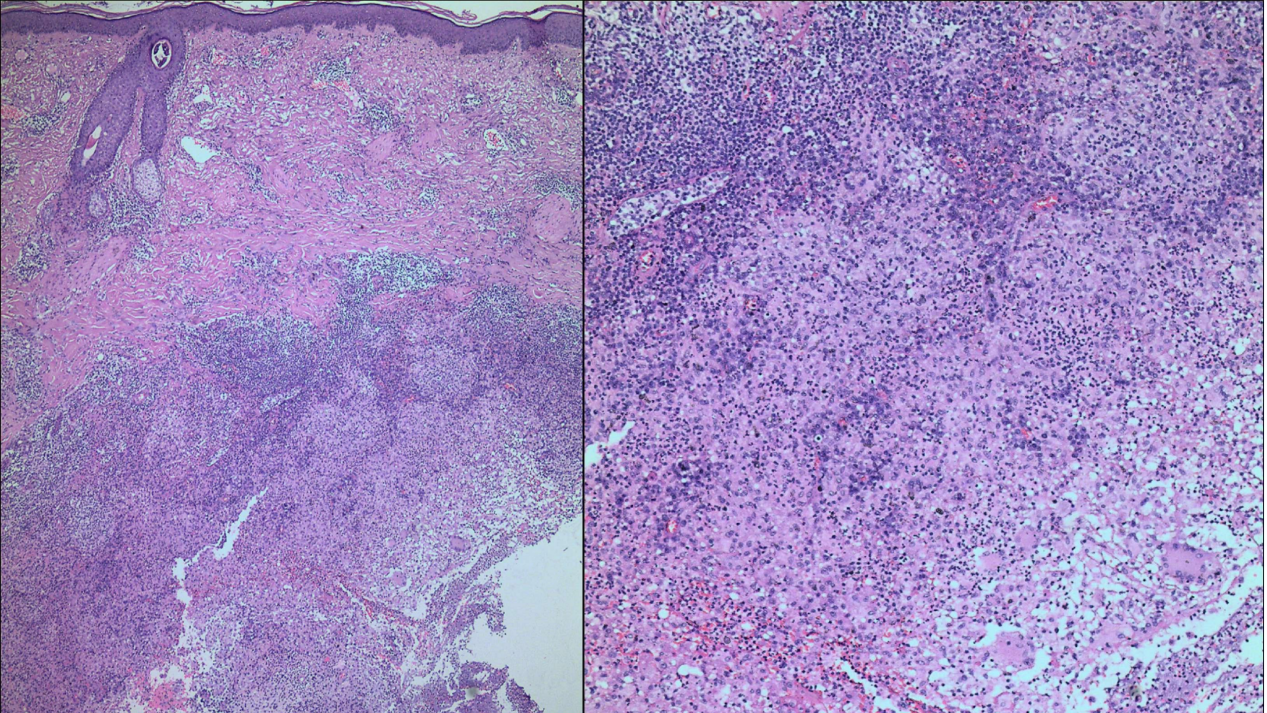


b

a

b

Fig. S2 a, b: Dermatopathological findings of the subcutaneous nodule at the right mandibular angle reveal a deep-seated diffuse mixed-cell infiltrate, including neutrophilic microabscesses, epithelioid cell granulomas, and significant infiltration of lymphocytes and multinucleated giant cells. (hematoxylin and eosin stain, a × 100, b × 400).
